# Safety of the Seasonal Influenza Vaccine in 2 Successive Pregnancies

**DOI:** 10.1001/jamanetworkopen.2024.34857

**Published:** 2024-09-19

**Authors:** Darios Getahun, In-Lu Amy Liu, Lina S. Sy, Jason M. Glanz, Ousseny Zerbo, Gabriela Vazquez-Benitez, Jennifer C. Nelson, Joshua T. Williams, Simon J. Hambidge, Huong Q. McLean, Stephanie A. Irving, Eric S. Weintraub, Lei Qian

**Affiliations:** 1 Department of Research & Evaluation, Kaiser Permanente Southern California, Pasadena; 2 Department of Health Systems Science, Kaiser Permanente Bernard J. Tyson School of Medicine, Pasadena, California; 3 Institute for Health Research, Kaiser Permanente Colorado, Denver; 4 Kaiser Permanente Vaccine Study Center, Kaiser Permanente Northern California, Oakland; 5 HealthPartners Institute, Minneapolis, Minnesota; 6 Kaiser Permanente Washington Health Research Institute, Seattle, Washington; 7 Denver Health Ambulatory Care Services, Denver, Colorado; 8 Marshfield Clinic Research Institute, Marshfield, Wisconsin; 9 Kaiser Permanente Center for Health Research, Portland, Oregon; 10 Immunization Safety Office, Centers for Disease Control and Prevention, Atlanta, Georgia

## Abstract

**Importance:**

Although influenza vaccination has been found to be safe in pregnancy, few studies have assessed repeated influenza vaccination over successive pregnancies, including 2 vaccinations in a year, in terms of adverse perinatal outcomes.

**Objective:**

To examine the association of seasonal influenza vaccination across successive pregnancies with adverse perinatal outcomes and whether the association varies by interpregnancy interval (IPI) and vaccine type (quadrivalent or trivalent).

**Design, Setting, and Participants:**

This retrospective cohort study included individuals with at least 2 successive singleton live-birth pregnancies between January 1, 2004, and December 31, 2018. Data were collected from the Vaccine Safety Datalink, a collaboration between the Centers for Disease Control and Prevention and integrated health care organizations. Data analysis was performed between January 8, 2021, and July 17, 2024.

**Exposures:**

Influenza vaccination was identified using vaccine administration codes. The vaccinated cohort consisted of people who received influenza vaccines during the influenza season (August 1 through April 30) in 2 successive pregnancies. The comparator cohort consisted of people identified as unvaccinated during both pregnancies.

**Main Outcomes and Measures:**

Main outcomes were risk of preeclampsia or eclampsia, placental abruption, fever, preterm birth, preterm premature rupture of membranes, chorioamnionitis, and small for gestational age among individuals with and without vaccination in both pregnancies. Adjusted relative risks (RRs) from Poisson regression were used to assess the magnitude of associations. The associations with adverse outcomes by IPI and vaccine type were evaluated.

**Results:**

Of 82 055 people with 2 singleton pregnancies between 2004 and 2018, 44 879 (54.7%) had influenza vaccination in successive pregnancies. Mean (SD) age at the start of the second pregnancy was 32.2 (4.6) years for vaccinated individuals and 31.2 (5.0) years for unvaccinated individuals. Compared with individuals not vaccinated in both pregnancies, vaccination in successive pregnancies was not associated with increased risk of preeclampsia or eclampsia (adjusted RR, 1.10; 95% CI, 0.99-1.21), placental abruption (adjusted RR, 1.01; 95% CI, 0.84-1.21), fever (adjusted RR, 0.87; 95% CI, 0.47-1.59), preterm birth (adjusted RR, 0.83; 95% CI, 0.78-0.89), preterm premature rupture of membranes (RR, 1.00; 95% CI, 0.94-1.06), chorioamnionitis (adjusted RR, 1.03; 95% CI, 0.90-1.18), or small for gestational age birth (adjusted RR, 0.99; 95% CI, 0.93-1.05). IPI and vaccine type did not modify the observed associations.

**Conclusions and Relevance:**

In this large cohort study of successive pregnancies, influenza vaccination was not associated with increased risk of adverse perinatal outcomes, irrespective of IPI and vaccine type. Findings support recommendations to vaccinate pregnant people or those who might be pregnant during the influenza season.

## Key Points

**Question:**

Is there an association between seasonal influenza vaccination across successive pregnancies and adverse perinatal outcomes, and is the association modified by interpregnancy interval (IPI) and vaccination type?

**Findings:**

In this cohort study of 82 055 people with 2 singleton pregnancies between 2004 and 2018, compared with individuals who were not vaccinated in both successive pregnancies, influenza vaccination in both successive pregnancies was not associated with an increased risk of adverse perinatal outcomes. IPI and vaccine type did not modify the findings.

**Meaning:**

These findings support the recommendations to vaccinate pregnant people or those who might become pregnant during the influenza season regardless of IPI and vaccine type.

## Teaser

This cohort study examines the association of seasonal influenza vaccination across successive pregnancies with adverse perinatal outcomes and whether the association varies by interpregnancy interval and vaccine type.

## Introduction

Seasonal influenza is an acute respiratory viral infection that can cause mild to severe illness with potentially life-threatening complications. It is a major public health issue that affects people of all ages, sexes, and racial and ethnic backgrounds. According to the World Health Organization, seasonal influenza affects 5% to 15% of the world’s population and results in up to 650 000 deaths annually.^1^ Although influenza is largely considered to be a self-limiting infection lasting 1 to 2 weeks, pregnant and postpartum people and their infants are especially vulnerable to influenza-related morbidity and mortality.^2,3,4,5,6,7^

Vaccination is the most effective method for preventing influenza infection and its related complications. The Advisory Committee on Immunization Practices of the Centers for Disease Control and Prevention (CDC)^8^ and the American College of Obstetrics and Gynecology^9^ recommend vaccinating everyone aged 6 months and older, including people who are or are planning to become pregnant, during the influenza season. In the US, influenza vaccination recommendations have been in place for pregnant people since the 1990s. Although recommendations and coverage among pregnant people have evolved, coverage was 47.2% during the 2022 to 2023 influenza season in the US.^10^ Influenza vaccination not only prevents influenza-related maternal morbidity,^11^ but its benefit also extends to newborns, as antibodies against the virus can pass from mothers to infants via the placenta or breastfeeding, protecting newborns from neonatal intensive care unit admissions,^11,12^ and against influenza and respiratory illnesses with fever for several months after birth, when the risk for developing these conditions is greatest.^13,14^

Although seasonal inactivated influenza A (pH1N1) vaccination during pregnancy has largely not been linked with adverse maternal and child outcomes,^11,15,16,17,18,19,20,21,22,23^ a case-control study suggested that influenza vaccination in people who received the pH1N1 influenza vaccine in a previous influenza season was associated with an increased risk for spontaneous abortion.^24^ However, a subsequent Vaccine Safety Datalink (VSD) follow-up case-control study reported no association between inactivated influenza vaccination and spontaneous abortion.^21^ Yet, its safety when given in successive pregnancies, including doses repeated within closely spaced successive pregnancies, has not previously been investigated, to our knowledge. Furthermore, the safety of the number of strains included in the influenza vaccine (trivalent vs quadrivalent) when given in successive pregnancies has not been studied, to our knowledge. Therefore, with the large number of pregnant people being vaccinated against seasonal influenza annually, the safety of repeated vaccination in successive pregnancies deserves closer investigation.

There are important considerations when studying vaccine safety in successive pregnancies. First, most adverse outcomes in a prior pregnancy, such as preterm birth and small for gestational age (SGA) birth, are strongly associated with recurrence in subsequent pregnancies. Therefore, studying adverse pregnancy outcomes requires accounting for potential risk factors for these adverse outcomes. Second, studies of the safety of routine vaccination during pregnancy also need to account for the role of potentially multiple exposures within short pregnancy intervals.^23,24^ Third, the safety profile may depend on the type of influenza vaccine (trivalent or quadrivalent) administered.

The objectives of this study were to assess whether seasonal influenza vaccination in successive pregnancies was associated with increased risk of adverse perinatal outcomes in the second pregnancy and whether estimated risks associated with vaccination were modified by the length of the interval between successive pregnancies and the vaccine type.

## Methods

This cohort study was approved by the institutional review boards of each participating site and the CDC with a waiver of informed consent per 45 C.F.R. part 46.101(c); 21 C.F.R. part 56. This study followed the Strengthening the Reporting of Observational Studies in Epidemiology (STROBE) reporting guideline.

### Study Population

This retrospective longitudinal cohort study included persons with successive singleton live-birth pregnancies delivered between 2004 and 2018 from 8 health care organizations participating in the VSD. The VSD project is a collaboration between the CDC and 13 integrated health care plans that uses large linked vaccination and electronic health record (EHR) databases to conduct postmarketing vaccine safety studies.^25^ The 8 participating VSD sites included Kaiser Permanente (KP) Southern California (lead site), Denver Health, HealthPartners Institute, KP Colorado, KP Northern California, KP Northwest, KP Washington, and Marshfield Clinic Research Institute.

From all 1 605 186 live births occurring at participating VSD sites during the study period, we excluded 366 220 pregnancies in the following categories: births at less than 20 weeks’ gestation, fetuses that weighed less than 500 g, multiple gestations, and pregnancies without documented prenatal care (eFigure 1 in Supplement 1). To be eligible, pregnant people must have had at least 2 successive singleton live-born pregnancies between 20 weeks’ and 42 weeks and 6 days’ gestation between January 1, 2004, and December 31, 2018, inclusive of continuous health plan membership (allowing for a 31-day gap) from pregnancy start date to pregnancy outcome date. Births at less than 20 weeks’ gestation were excluded to avoid errors in gestational age estimation of births at borderline viability. Furthermore, we excluded 201 people who had received an influenza vaccinations between May 1 through July 31, which is outside the influenza vaccination season (August 1 through April 30). After these exclusions, we had a cohort of 125 830 people with 251 660 consecutive pregnancies (44 879 individuals [36%] vaccinated in both pregnancies and 37 176 individuals [30%] unvaccinated in both pregnancies). The study had sufficient statistical power to detect a relative risk (RR) of 1.1 for an outcome that had a rate of 0.06 per pregnancy (eTable 1 in Supplement 1). We excluded individuals with only 1 vaccination during their successive pregnancies (43 775 individuals). For individuals with more than 2 successive pregnancies during the study period, only their first 2 successive pregnancies were included in the analysis. The study cohort composition is shown in eFigure 1 in Supplement 1.

### Exposure Measures

Influenza vaccination was identified using vaccine administration codes. VSD immunization records include vaccines administered within participating sites and vaccines from claims data, member self-reports with documentation, or the state immunization registry, if available. A pregnant person who received a documented influenza vaccine during the influenza season was considered vaccinated for that season. For this analysis, vaccination during pregnancy was defined as receipt of a seasonal influenza vaccine from 30 days before the date of conception until the end of pregnancy. The vaccinated cohort included pregnant people who received influenza vaccines in 2 successive pregnancies. The index date was the vaccination date of the second (ie, latter) pregnancy. The comparator cohort consisted of people who were unvaccinated during both pregnancies. An index date was assigned during their second pregnancy based on the distribution of gestational age in the respective week of vaccinated people on their index date.

### Outcome Measures

*International Classification of Diseases, Ninth Revision, Clinical Modification (ICD-9-CM)* and *International Statistical Classification of Diseases, Tenth Revision, Clinical Modification (ICD-10-CM) *diagnosis and procedure codes (eTable 2 in Supplement 1), maternal temperature, gestational age, and birth weight were used to ascertain outcomes. Maternal and infant health outcomes in the vaccinated and unvaccinated cohorts were assessed for the second pregnancy of each participant during the follow-up period. The outcomes of interest were maternal fever (>100.4 °F or with a fever diagnosis) first reported within 7 days after vaccination during the second pregnancy (or index date in unvaccinated pregnant people),^26,27^ preterm birth (birth at <37 weeks’ gestation) and its subtypes (spontaneous and iatrogenic), preterm premature rupture of membranes (PPROM), chorioamnionitis, SGA birth, preeclampsia or eclampsia, and placental abruption. Medically indicated (iatrogenic) preterm birth was defined as preterm birth with an accompanying procedure or diagnosis code for induction of labor or overall cesarean deliveries, which are listed in eTable 2 in Supplement 1. We used a widely used criterion to define SGA as birth weight: below the tenth percentile based on race-, ethnicity-, and sex-specific birth weight for gestational age cutoffs (internal standard) for all live births in the VSD sites.^28,29^

### Covariates

The longitudinally linked successive pregnancy records extracted from the EHR contained information on self-reported race and ethnicity, age at pregnancy start, behavioral factors (timing of prenatal care initiation, number of prenatal care visits, smoking and alcohol use during pregnancy) based on the start date of the index pregnancy, maternal comorbidities (chronic hypertension, diabetes, kidney and autoimmune disease) recorded any time before the second pregnancy conception date, history of prespecified adverse perinatal outcomes in a prior pregnancy, timing of conception, and calendar year of pregnancy. Race and ethnicity were assessed because they are potential confounders. Other variables examined were the interpregnancy interval (IPI; the interval between a birth and a subsequent pregnancy), influenza vaccination type (trivalent or quadrivalent), receipt of influenza vaccine during the IPI, prepregnancy body mass index (BMI) measured close to the date of conception, gestational weight gain according to the Institute of Medicine guidelines,^30^ receipt of other vaccines during pregnancy, and VSD site. When data on prepregnancy BMI were not available, BMI measured at first visit during the first trimester of pregnancy was used. Data on maternal educational attainment came from birth certificate records.

### Statistical Analysis

We compared the distribution of maternal demographic, behavioral, and baseline clinical characteristics between vaccinated and unvaccinated cohorts. Continuous variables were summarized by mean and SD and compared using *t* tests; categorical data were summarized by frequency and percentage and compared using the χ^2^ test. Differences with *P* < .05 were considered statistically significant, and all tests were 2-sided.

We calculated crude incidence for each outcome for vaccinated and unvaccinated cohorts. The incidence consisted of the number of people or infants with the condition in the numerator and the total number of people or infants in the denominator.

Relative risks (RRs) with 95% CIs for the association between influenza vaccination and each outcome were estimated by Poisson regression models with and without adjustment for history of adverse perinatal outcomes in a prior pregnancy (yes or no) and potential confounders in the second pregnancy, including maternal age at second pregnancy start (<20, 20-24, 25-29, 30-34, and ≥35 years), self-reported race and ethnicity in mutually exclusive categories (Hispanic, non-Hispanic Asian, non-Hispanic Black, non-Hispanic White, multiple or other [including multiple races, Native American, and Pacific Islander], and unknown), highest level of educational attainment (≤12th grade, high school graduate, some college, college with degree, or unknown), smoking and alcohol use during pregnancy (yes, no, or unknown or missing), prepregnancy BMI (continuous), gestational weight gain according to the Institute of Medicine guidelines (continuous),^30^ timing of prenatal care initiation (early [≤3 months] or late [>3 months]), number of prenatal care visits (continuous), maternal comorbidities (chronic hypertension, diabetes, kidney disease, and autoimmune disease), calendar month of conception, and calendar year of pregnancy. Other variables adjusted for in the model included the IPI (<1.00, 1.00-1.49, 1.50-1.99, 2.00-2.49, 2.50-2.99, 3.00-3.49, and ≥3.50 years), receipt of influenza vaccine during the IPI (yes or no), receipt of other vaccines during the second pregnancy (yes or no), and VSD site. Tests for interaction were performed between vaccination status and IPI. Furthermore, we performed frequency matching of gestational age by assigning the index (vaccination) date of vaccinated individuals to unvaccinated individuals. A Poisson regression model was used to estimate the RR. For common outcomes (ie, incidence >2%), a Poisson regression model with a robust variance was used. For events identified any time during the pregnancy after the index date (ie, preeclampsia or eclampsia, placental abruption, PPROM, and chorioamnionitis), the analysis also accounted for follow-up person-time. Follow-up began on the index date and ended on the date of each outcome or end of the pregnancy, whichever came first.

We examined the association between influenza vaccine and adverse perinatal outcomes in successive pregnancies stratified by IPI (<1.0, 1.0-2.9, and ≥3.0 years). Lastly, a stratified analysis was conducted to investigate the association between adverse perinatal outcomes in the second pregnancy among people vaccinated and unvaccinated for influenza in successive pregnancies by vaccine type. The analyses were performed between January 8, 2021, and July 17, 2024, using SAS software version 9.4 (SAS Institute).

## Results

After exclusions, the total cohort included 82 055 people with the same vaccination status in 2 singleton pregnancies. Of these, 44 879 individuals (54.69%) had influenza vaccination in successive pregnancies, and 37 176 individuals (45.31%) unvaccinated in both pregnancies. The mean (SD) age at the start of the second pregnancy was 32.22 (4.62) years for vaccinated individuals and 31.17 (5.01) years for unvaccinated individuals. Table 1 shows the distribution of maternal characteristics in the second pregnancy based on influenza vaccination status in both pregnancies. Compared with people who were not vaccinated in either pregnancy, people who were vaccinated in both successive pregnancies were more likely to be aged 30 years or older (Table 1). Among people who were not vaccinated in either pregnancy, 12 601 (33.90%) were Hispanic, 5171 (13.91%) were non-Hispanic Asian, 2568 (6.91%) were non-Hispanic Black, 14 220 (38.25%) were non-Hispanic White, and 1827 (4.91%) identified as multiple or other races; whereas among individuals who were vaccinated during both pregnancies, 12 506 (27.87%) were Hispanic, 9164 (20.42%) were non-Hispanic Asian, 1286 (2.87%) were non-Hispanic Black, 19 475 (43.39%) were non-Hispanic White, and 2033 (4.53%) identified as multiple or other races. Individuals who were vaccinated during both pregnancies also tended to have graduated from college with a degree, received more frequent prenatal care, initiated prenatal care early, not smoked or consumed alcohol during pregnancy, had an IPI between 1.00 and 3.49 years, had 1 or more comorbidities, received influenza vaccine during the IPI, and received a noninfluenza vaccine during the second pregnancy (Table 1). Of vaccinated individuals, a relatively large proportion (15 154 individuals [33.77%]) were vaccinated in the first trimester of pregnancy. Distributions by gestational week at time of index date in the influenza vaccinated and unvaccinated groups were similar (eFigure 2 in Supplement 1).

**Table 1.** Maternal Characteristics at Second Pregnancy by Influenza Vaccination Status

Characteristics	Individuals, No. (%)			*P* value
	Total pregnancies (N = 82 055)	Influenza vaccination		
		Not vaccinated in both pregnancies (n = 37 176)	Vaccinated in both pregnancies (n = 44 879)	
Maternal age, y				
Mean (SD)	31.75 (4.83)	31.17 (5.01)	32.22 (4.62)	<.001
<20	964 (1.17)	580 (1.56)	384 (0.86)	<.001
20-24	6723 (8.19)	3800 (10.22)	2923 (6.51)	
25-29	19 279 (23.50)	10 026 (26.97)	9253 (20.62)	
30-34	34 059 (41.51)	14 242 (38.31)	19 817 (44.16)	
≥35	21 030 (25.63)	8528 (22.94)	12 502 (27.86)	
Maternal race and ethnicity				
Hispanic	25 107 (30.60)	12 601 (33.90)	12 506 (27.87)	<.001
Non-Hispanic Asian	14 335 (17.47)	5171 (13.91)	9164 (20.42)	
Non-Hispanic Black	3854 (4.70)	2568 (6.91)	1286 (2.87)	
Non-Hispanic White	33 695 (41.06)	14 220 (38.25)	19 475 (43.39)	
Multiple/other^a^	3860 (4.70)	1827 (4.91)	2033 (4.53)	
Missing	1204 (1.47)	789 (2.12)	415 (0.92)	
Maternal education				
≤12th Grade	1627 (1.98)	945 (2.54)	682 (1.52)	<.001
High school graduate	7857 (9.58)	4262 (11.46)	3595 (8.01)	
Some college	28 826 (35.13)	14 482 (38.96)	14 344 (31.96)	
College with degree	36 267 (44.20)	14 584 (39.23)	21 683 (48.31)	
Unknown	7478 (9.11)	2903 (7.81)	4575 (10.19)	
PNC visits, mean (SD), No.	12.59 (4.95)	12.13 (4.69)	12.97 (5.12)	<.001
Early (≤3 mo) initiation of PNC	79 273 (96.60)	35 449 (95.35)	43 824 (97.65)	<.001
Smoking during pregnancy	5166 (6.30)	2638 (7.10)	2528 (5.63)	<.001
Alcohol use during pregnancy	14 421 (17.57)	5776 (15.54)	8645 (19.26)	<.001
IPI				
Mean (SD), d	765.64 (557.36)	783.47 (612.04)	750.87 (507.16)	<.001
Category, y				
<1.00	16 927 (20.63)	8552 (23.00)	8375 (18.66)	<.001
1.00-1.49	18 458 (22.49)	7994 (21.50)	10 464 (23.32)	
1.50-1.99	13 906 (16.95)	5749 (15.46)	8157 (18.18)	
2.00-2.49	10 556 (12.86)	4405 (11.85)	6151 (13.71)	
2.50-2.99	6340 (7.73)	2658 (7.15)	3682 (8.20)	
3.00-3.49	4548 (5.54)	2055 (5.53)	2493 (5.55)	
≥3.50	11 320 (13.80)	5763 (15.50)	5557 (12.38)	
Gestational age at index date, trimester				
First^b^	34 280 (41.78)	15 554 (41.84)	18 726 (41.73)	.95
Second	24 608 (29.99)	11 136 (29.95)	13 472 (30.02)	
Third	23 167 (28.23)	10 486 (28.21)	12 681 (28.26)	
Gestational weight gain, mean (SD), lbs	27.30 (13.56)	27.29 (14.05)	27.30 (13.17)	.57
Prepregnancy BMI, mean (SD)	26.44 (6.15)	26.54 (6.14)	26.36 (6.15)	<.001
Maternal comorbidities				
Chronic hypertension	4352 (5.3)	1913 (5.15)	2439 (5.43)	.07
Pregestational diabetes	1690 (2.06)	726 (1.95)	964 (2.15)	.05
Kidney disease	5615 (6.84)	2559 (6.88)	3056 (6.81)	.68
Autoimmune disease	6020 (7.34)	2567 (6.90)	3453 (7.69)	<.001
Vaccine formulation				
Trivalent	NA	NA	25 863 (57.63)	NA
Quadrivalent	NA	NA	8282 (18.45)	NA
Other	NA	NA	10 734 (23.92)	NA
Receipt of influenza vaccination during the IPI	39 535 (48.18)	9531 (25.64)	30 004 (66.86)	<.001
Receipt of a noninfluenza vaccine in second pregnancy	7511 (9.15)	2268 (6.10)	5243 (11.68)	<.001

Abbreviations: BMI, body mass index (calculated as weight in kilograms divided by height in meters squared); IPI, interpregnancy interval; NA, not applicable; PNC, prenatal care.

^a^ Multiple/other groups captured all other race/ethnicity categories including multiple races, Native American, and Pacific Islander.

^b^ Including index (vaccination) dates during the 30 days before conception.

Compared with people who were not vaccinated in successive pregnancies, those vaccinated in successive pregnancies had a higher incidence of preeclampsia or eclampsia (2.95 events vs 3.39 events per 100 pregnancies), PPROM (8.41 events vs 9.51 events per 100 pregnancies), and chorioamnionitis (1.69 events vs 1.93 events per 100 pregnancies) (Figure; eTable 3 in Supplement 1). In crude analysis, compared with being unvaccinated in successive pregnancies, vaccination in successive pregnancies was associated with an increased risk of preeclampsia or eclampsia (RR, 1.15; 95% CI, 1.06-1.24), PPROM (RR, 1.13; 95% CI, 1.08-1.18), and chorioamnionitis (RR, 1.14; 95% CI, 1.03-1.26) (eTable 3 in Supplement 1). However, after adjustment for potential confounding factors, vaccination in successive pregnancies was no longer an independent risk factor associated with the development of any of the outcomes under investigation (Figure; eTable 3 in Supplement 1). In adjusted analyses, we found that pregnant people who had (vs had not) been vaccinated in successive pregnancies were not at an increased risk of placental abruption (adjusted RR, 1.01; 95% CI, 0.84-1.21), maternal fever (adjusted RR, 0.87; 95% CI, 0.47-1.59), preterm birth (adjusted RR, 0.83; 95% CI, 0.78-0.89), or SGA birth (adjusted RR, 0.99; 95% CI, 0.93-1.05).

**Figure.**
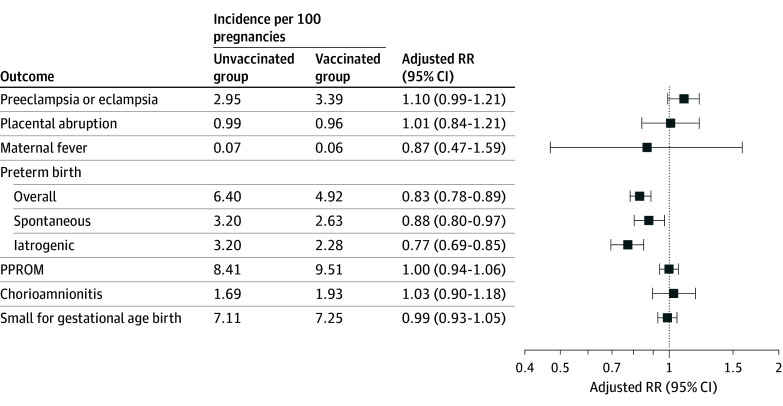
Outcome Incidence Based on Maternal Vaccination Status in Successive Pregnancies and Adjusted Relative Risk (RR) of Adverse Perinatal Outcomes in Second Pregnancy The vaccinated group included individuals who were vaccinated with a seasonal influenza vaccine in 2 successive pregnancies, and the unvaccinated group was individuals who were not vaccinated with influenza vaccine in 2 successive pregnancies. Analyses were adjusted for maternal age, race and ethnicity, maternal education, smoking and alcohol use during pregnancy, prepregnancy body mass index, gestational weight gain, timing of prenatal care initiation and number of prenatal care visits, maternal comorbidities (chronic hypertension, diabetes, kidney disease, and autoimmune disease), history of adverse perinatal outcomes in a prior pregnancy, month of conception, year of pregnancy, interpregnancy interval, receipt of influenza vaccine during the interpregnancy period, receipt of other vaccines during pregnancy, and Vaccine Safety Datalink site. PPROM indicates preterm premature rupture of membranes.

No statistical interaction was detected between vaccination status and IPI (eTable 4 in Supplement 1). Therefore, the risk of adverse perinatal outcomes was not different between vaccinated and unvaccinated for any of the strata of IPI (Table 2). Furthermore, analysis of influenza vaccine type revealed that receipt of either quadrivalent or trivalent influenza vaccine during successive pregnancies did not modify the association between influenza vaccination and perinatal outcomes (eTable 5 in Supplement 1).

**Table 2.** Incidence and aRR of Adverse Outcomes in Second Pregnancy Among People With Influenza Vaccination in Successive Pregnancies vs People With No Influenza Vaccination in Successive Pregnancies, Stratified by Interpregnancy Interval

Adverse outcome in the second pregnancy	IPI <1 y			IPI 1.00-2.99 y			IPI ≥3 y		
	Incidence per 100 pregnancies		aRR (95% CI)^a^	Incidence per 100 pregnancies		aRR (95% CI)^a^	Incidence per 100 pregnancies		aRR (95% CI)^a^
	Not vaccinated (n = 8552)	Vaccinated (n = 8375)		Not vaccinated (n = 20 806)	Vaccinated (n = 28 454)		Not vaccinated (n = 7818)	Vaccinated (n = 8050)	
Preeclampsia or eclampsia	2.47	3.25	1.20 (0.97-1.47)	2.60	3.02	1.04 (0.91-1.19)	4.43	4.86	0.93 (0.78-1.11)
Placental abruption	1.04	1.16	1.04 (0.73-1.48)	0.91	0.84	0.96 (0.75-1.23)	1.16	1.16	1.04 (0.72-1.50)
Maternal fever	0.07	0.11	NA^b^	0.07	0.05	0.63 (0.29-1.36)	0.05	0.06	NA^b^
Preterm birth									
Any	7.43	5.42	0.85 (0.75-0.98)	5.49	4.50	0.87 (0.79-0.96)	7.69	5.88	0.76 (0.66-0.88)
Spontaneous preterm birth	4.00	2.94	0.90 (0.75-1.08)	2.76	2.49	0.92 (0.80-1.06)	3.48	2.83	0.78 (0.64-0.97)
Iatrogenic preterm birth	3.43	2.48	0.79 (0.64-0.97)	2.73	2.01	0.80 (0.69-0.93)	4.21	3.04	0.72 (0.59-0.88)
PPROM	6.08	7.34	0.96 (0.84-1.10)	8.13	9.54	0.97 (0.90-1.04)	11.70	11.68	0.90 (0.81-1.00)
Chorioamnionitis	1.30	1.56	1.18 (0.86-1.60)	1.66	1.81	0.91 (0.77-1.08)	2.17	2.72	1.12 (0.88-1.43)
SGA birth	6.74	6.47	0.99 (0.87-1.13)	7.00	7.14	0.98 (0.91-1.06)	7.84	8.43	1.03 (0.91-1.17)

Abbreviations: aRR, adjusted relative risk; NA, not available; PPROM, preterm premature rupture of membranes; SGA, small for gestational age.

^a^ Analyses were adjusted for maternal age, race and ethnicity, maternal education, smoking, and alcohol use during pregnancy, prepregnancy body mass index, gestational age at index date, gestational weight gain, timing of prenatal care initiation and number of prenatal care visits, maternal comorbidities (chronic hypertension, diabetes, kidney disease, and autoimmune disease), history of adverse perinatal outcomes in a prior pregnancy, month of conception, year of pregnancy, receipt of influenza vaccine during the interpregnancy period, receipt of other vaccines during pregnancy, and Vaccine Safety Datalink site.

^b^ Estimate was not available due to small sample size and model convergence issue.

## Discussion

In this cohort study using a large, demographically and geographically diverse sample of pregnant people, we observed that receipt of influenza vaccination in 2 successive pregnancies was not associated with increased risk of preeclampsia or eclampsia, placental abruption, maternal fever, preterm birth (spontaneous and iatrogenic), PPROM, chorioamnionitis, or SGA birth compared with individuals who were unvaccinated in 2 successive pregnancies. The risk of adverse perinatal outcomes in successive pregnancies was not significantly different between vaccinated and unvaccinated individuals for any strata of the interval between 2 consecutive pregnancies. We also observed no association of receiving trivalent or quadrivalent influenza vaccine types during successive pregnancies with adverse perinatal outcomes of interest.

Several studies have examined the safety of influenza vaccination during pregnancy^12,13,14,15,16,17,18,19,20,21^; however, to our knowledge, longitudinal studies have not been conducted to evaluate safety when influenza vaccines are administered in successive pregnancies, especially within short IPI. Furthermore, the association between trivalent or quadrivalent influenza vaccines administered repeatedly in successive pregnancies and adverse perinatal outcomes has not been studied, to our knowledge. Therefore, this study assessed the safety of influenza vaccination during successive pregnancies from automated EHR data compiled from 8 geographically diverse integrated health care organizations across the US. We hypothesized that vaccination against seasonal influenza in successive pregnancies was not associated with increased risk of adverse perinatal outcomes, even among individuals who were conceiving over intervals of less than 1 year.

In a case-control study using VSD data from 2 influenza seasons (2010-2011 and 2011-2012), Donahue et al^24^ examined the association between influenza vaccination and risk of spontaneous abortion. Although the study by Donahue et al^24^ was not designed to investigate the safety of repeated influenza vaccination in successive pregnancies, their findings suggested influenza vaccination was statistically significantly associated with a risk of spontaneous abortion only in individuals who were vaccinated with pH1N1-containing influenza vaccine in the previous influenza season (adjusted odds ratio, 7.7; 95% CI, 2.2-27.3). This finding from Donahue et al^24^ prompted subsequent studies to further investigate the potential association. However, in a larger VSD follow-up study, influenza vaccination was not associated with risk of spontaneous abortion during the 2012 to 2013, 2013 to 2014, or 2014 to 2015 seasons, irrespective of previous season vaccination.^21^ Aligned with our hypothesis, we found no association of adverse perinatal outcomes among people who received seasonal influenza vaccine in successive pregnancies ending in live births. Additional analysis stratified by length of IPIs and vaccine type revealed that irrespective of IPIs and vaccine type, people who had (vs had not) been vaccinated in both pregnancies had no increased risk of adverse perinatal outcomes.

To our knowledge, this is the first study to assess the safety of seasonal influenza vaccination when given in 2 successive pregnancies and whether a shorter IPI was associated with modified risk. Furthermore, this study was the first to our knowledge to investigate the association between vaccine types and adverse perinatal outcomes in successive pregnancies, providing further evidence that the number of strains included in the influenza vaccine (trivalent vs quadrivalent) was not associated with the outcomes of interest. Safety data on influenza vaccination during the first trimester of pregnancy remain limited. In this study, more than one-third of the vaccinated group of pregnant individuals were vaccinated during their first trimester of pregnancy. Therefore, research using a large population-based database, such as the VSD, can provide much-needed safety data for first trimester vaccination.

### Strengths and Limitations

Strengths of this study include the use of the VSD’s large population-based EHR dataset compiled from health care organizations reflecting the demographic diversity of the entire US (approximately 3% of the population). The VSD permits longitudinal linkage to study successive pregnancy outcomes with relatively accurate and complete inpatient and outpatient health outcome information and vaccination status. A further strength of our study is the ability to control for several potential confounding factors. Adjusting for history of adverse outcomes in a prior pregnancy and frequency matching of gestational age by assigning the index (vaccination) date of vaccinated people to unvaccinated people are additional strengths of this study; both are critical factors to account for when examining the safety of vaccination in successive pregnancies.

This study also has some limitations. There is the potential for vaccination (exposure) misclassification due to receipt of influenza vaccination outside the participating health care organizations (eg, workplace or retail pharmacy). Nevertheless, in a VSD survey study of pregnant people, EHR-based influenza vaccination data were generally concordant with self-report.^31^ Another potential limitation is we only included pregnancies resulting in live births, since gestational age dating information was more reliable and data on outcomes of interest were more complete. We consider findings from this study generalizable to successive singleton live-born pregnancies. Although we attempted to minimize confounding by adjusting for a history of outcomes in a prior pregnancy and matching the analysis by gestational age at index date, as with any observational study, our results may be subject to bias due to potential unmeasured confounders.

## Conclusions

The findings of this large, retrospective longitudinal cohort study suggest that influenza vaccination in successive pregnancies was not associated with an increased risk of prespecified adverse perinatal outcomes, even if administered over intervals of less than 1 year. Our results have important implications for health care practitioners and people having successive pregnancies. These are reassuring results regarding influenza vaccine safety for pregnant people, in that influenza vaccination in successive pregnancies, even within short IPIs, is not associated with increased risk of adverse perinatal outcomes. The study findings support recommendations to vaccinate people during pregnancy regardless of the interval between any 2 successive pregnancies and the type of vaccination.

## Notes

Supplement 1. **eTable 1.** Total Sample Size Required to Achieve 80% Power for Various Detectable Relative Risks and Outcome Rates, Assuming the Sizes of the Vaccinated and Unvaccinated Groups Are Equal and a Type 1 Error Rate of 0.05. **eTable 2. **List of Outcomes of Interest and Corresponding *ICD-9*/*ICD-10* Codes **eTable 3. **Rates and Relative Risks of Adverse Outcomes in Second Pregnancy Based on Maternal Vaccination Status in Successive Pregnancies **eTable 4. **Test for Interaction Between Vaccination Status and Interpregnancy Interval **eTable 5. **Incidence and Relative Risk of Adverse Perinatal Outcomes in Second Pregnancy Among People With Influenza Vaccination in Successive Pregnancies Versus People With No Influenza Vaccination in Successive Pregnancies, by Vaccine Type: Vaccine Safety Datalink, 2004-2018 **eFigure 1.** Cohort Composition: Vaccine Safety Datalink, 2004-2018 **eFigure 2.** Distribution of Gestational Week at Index Date in the Influenza Vaccinated Group and Unvaccinated Group: Vaccine Safety Datalink, 2004-2018

Supplement 2.
Data Sharing Statement
