# Supplementary material for: Safety of the Seasonal Influenza Vaccine in 2 Successive Pregnancies
Source: JAMA Netw Open. 2024 Sep 19;7(9):e2434857. doi: 10.1001/jamanetworkopen.2024.34857 (PMC11413712; doi:10.1001/jamanetworkopen.2024.34857)

Supplemental Online Content

Getahun D, Liu IA, Sy LS, et al. Safety of the seasonal influenza vaccine in 2 successive pregnancies. *JAMA Netw Open*. 2024;7(9):e2434857. doi:10.1001/jamanetworkopen.2024.34857

eTable 1. Total Sample Size Required to Achieve 80% Power for Various Detectable Relative Risks and Outcome Rates, Assuming the Sizes of the Vaccinated and Unvaccinated Groups Are Equal and a Type 1 Error Rate of 0.05.
eTable 2. List of Outcomes of Interest and Corresponding *ICD-9*/*ICD-10* Codes
eTable 3. Rates and Relative Risks of Adverse Outcomes in Second Pregnancy Based on Maternal Vaccination Status in Successive Pregnancies
eTable 4. Test for Interaction Between Vaccination Status and Interpregnancy Interval
eTable 5. Incidence and Relative Risk of Adverse Perinatal Outcomes in Second Pregnancy Among People With Influenza Vaccination in Successive Pregnancies Versus People With No Influenza Vaccination in Successive Pregnancies, by Vaccine Type
eFigure 1. Cohort Composition: Vaccine Safety Datalink (VSD), 2004-2018
eFigure 2. Distribution of Gestational Week at Index Date in the Influenza Vaccinated Group and Unvaccinated Group: Vaccine Safety Datalink, 2004-2018

This supplemental material has been provided by the authors to give readers additional information about their work.

© 2024 Getahun D et al. *JAMA Network Open.*

eTable 1. Total sample size required to achieve 80% power for various detectable relative risks and outcome rates, assuming the sizes of the vaccinated and unvaccinated groups are equal and a type 1 error rate of 0.05.

Outcome rate in unvaccinated group	Relative risk	Total sample size
0.06	1.1	51,480
	1.2	13,438
	1.5	2,418
	1.8	1,048
0.03	1.1	106,422
	1.2	27,828
	1.5	5,034
	1.8	2,192
0.01	1.1	326,190
	1.2	85,386
	1.5	15,500
	1.8	6,770
0.001	1.1	3,293,058
	1.2	862,424
	1.5	156,780
	1.8	68,580

© 2024 Getahun D et al. *JAMA Network Open.*

eTable 2: List of outcomes of interest and corresponding ICD-9/ICD-10 codes

Outcome	ICD-9 codes	ICD-10 codes
Preterm premature rupture of membranes	658.1, 658.10, 658.11, 658.13, 658.20, 658.21, 761.1	O42.0, O42.00, O42.01, O42.010, O42.011, O42.012, O42.013, O42.019, O42.02, O42.1, O42.10, O42.11, O42.111, O42.112, O42.113, O42.119, O42.12, O42.9, O42.90, O42.91, O42.911, O42.912, O42.913, O42.919, O42.92, P01.1
Chorioamnionitis	762.7, 658.4, 658.40, 658.41, 658.43	P02.7, O41.1090, O41.1290, O41.1490, O41.1010, O41.1020, O41.1030, O41.1210, O41.1220, O41.1230, O41.1410, O41.1420, O41.1430
Preeclampsia	642.40, 642.41, 642.42, 642.43, 642.44, 642.50, 642.51, 642.52, 642.53, 642.54, 642.70, 642.71, 642.72, 642.73, 642.74	O11.1, O11.2, O11.3, O11.4, O11.5, O11.9, O14.0, O14.00, O14.02, O14.03, O14.04, O14.05, O14.1, O14.10, O14.12, O14.13, O14.14, O14.15, O14.20, O14.22, O14.23, O14.24, O14.25, O14.9, O14.90, O14.92, O14.93, O14.94, O14.95
Eclampsia	642.60, 642.61, 642.62, 642.63, 642.64	O15.0, O15.00, O15.02, O15.03, O15.1, O15.2, O15.9
Placental abruption	762.1, 641.20, 641.21, 641.23	P02.1, O45.8, O45.8X1, O45.8X2, O45.8X3, O45.8X9, O45.0, O45.00, O45.001, O45.002, O45.003, O45.009, O45.01, O45.011, O45.012, O45.013, O45.019, O45.02, O45.021, O45.022, O45.023, O45.029, O45.09, O45.091, O45.092, O45.093, O45.099, O45.009, O45.9, O45.90, O45.91, O45.92, O45.93
Induction of labor	DXCODE 659.00, 659.01, 659.03, 659.10, 659.11, 659.13<br>OR<br>PXCODE 73.01, 73.1, 73.4	DXCODE O61.0, O61.1, O61.8, O61.9<br>OR<br>PXCODE 0U7C7ZZ
Overall Cesarean deliveries	DXCODE 674.10, 674.12, 674.14, 669.7,669.70, 669.71, 763.4<br>OR<br>PXCODE 74.0, 74.1, 74.2, 74.4, 74.9, 74.99	DXCODE V30.01, V31.01, V32.01, V33.01, V34.01, V35.01, V36.01, V37.01, V39.01, O66.4, O66.5, O90.0, O82, O82.0, O82.1, O82.2, O82.8, O82.9, P03.4, Z38.01, Z38.31, Z38.68, Z38.69<br>OR<br>PXCODE 10D00Z0, 10D00Z1, 10D00Z2
Maternal fever	659.2, 659.21, 659.23	O75.2

ICD-9/ICD-10 codes were identified from all care settings except for virtual settings.

© 2024 Getahun D et al. *JAMA Network Open.*

eTable 3 . Rates and relative risks of adverse outcomes in 2nd pregnancy based on maternal vaccination status in successive pregnancies.

**Outcomes in the 2nd pregnancy**	**Second pregnancy**			
	**Rate of adverse events**		**Relative risk (95% confidence interval)[a]**	
	**Unvaccinated in both pregnancies (%) N= 37,176**	**Vaccinated in both pregnancies (%) N= 44,879**	**Unadjusted**	**Adjusted**
Preeclampsia/eclampsia	2.95	3.39	**1.15 (1.06, 1.24)**	1.10 (0.99, 1.21)
Placental abruption	0.99	0.96	0.96 (0.84, 1.11)	1.01 (0.84, 1.21)
Maternal fever (>100.4˚F)	0.07	0.06	0.96 (0.56, 1.64)	0.87 (0.47, 1.59)
Preterm birth (PTB)	6.4	4.92	0.77 (0.73, 0.81)	0.83 (0.78, 0.89)
Spontaneous PTB	3.2	2.63	0.82 (0.76, 0.89)	0.88 (0.80, 0.97)
Iatrogenic PTB	3.2	2.28	0.71 (0.66, 0.77)	0.77 (0.69, 0.85)
Preterm PROM	8.41	9.51	**1.13 (1.08, 1.18)**	1.00 (0.94, 1.06)
Chorioamnionitis	1.69	1.93	**1.14 (1.03, 1.26)**	1.03 (0.90, 1.18)
Small for gestational age birth	7.11	7.25	1.02 (0.97, 1.07)	0.99 (0.93, 1.05)

Abbreviations: PROM, premature rupture of membrane

[a] Analyses were adjusted for maternal age, race and ethnicity, maternal education, smoking, and alcohol use during pregnancy, pre-pregnancy body mass index, gestational weight gain, timing of prenatal care initiation/number of prenatal care visits, maternal comorbidities (chronic hypertension, diabetes mellitus, renal disease, and autoimmune disease), history of adverse perinatal outcomes in a prior pregnancy, month of conception, year of pregnancy, interpregnancy interval, receipt of influenza vaccine during the interpregnancy period, receipt of other vaccines during pregnancy, and Vaccine Safety Datalink site.

© 2024 Getahun D et al. *JAMA Network Open.*

eTable 4. Test for interaction between vaccination status and interpregnancy interval (IPI).

Outcomes in the 2nd pregnancy	P-value for testing vaccination status and IPI interaction*
Preeclampsia/eclampsia	0.52
Placental abruption	0.91
Maternal fever	0.76
Preterm birth (PTB)	0.48
Spontaneous PTB	0.44
Iatrogenic PTB	0.71
Preterm PROM	0.15
Chorioamnionitis	0.52
Small for gestational age birth	0.79

Abbreviations: PROM, premature rupture of membranes
*Adjusted type 3 p-values.

© 2024 Getahun D et al. *JAMA Network Open.*

eTable 5 Incidence (per 100 pregnancies) and relative risk of adverse perinatal outcomes in second pregnancy among people with influenza vaccination in successive pregnancies versus people with no influenza vaccination in successive pregnancies, by vaccine type (quadrivalent or trivalent): Vaccine Safety Datalink, 2004-2018

Adverse outcomes in 2nd pregnancy	Quadrivalent			Trivalent		
	Incidence		Adjusted RR (95% CI)[a]	Incidence		Adjusted RR (95% CI)[a]
	No vac N=34,176	Vac N=8,282		No vac N=34,176	Vac N=25,863	
PE/eclampsia	2.92	3.97	1.00 (0.84, 1.19)	2.92	3.19	0.97 (0.86, 1.08)
Placental abruption	0.99	1.03	1.07 (0.77, 1.49)	0.99	0.89	0.94 (0.76, 1.16)
Maternal fever	0.07	0.12	0.97 (0.39, 2.41)	0.07	0.07	N/A[b]
Preterm birth (PTB)	6.27	4.78	0.79 (0.68, 0.91)	6.27	4.96	0.89 (0.82, 0.97)
Spontaneous PTB	3.14	2.64	0.84 (0.69, 1.02)	3.14	2.69	0.96 (0.86, 1.07)
Iatrogenic PTB	3.13	2.14	0.71 (0.58, 0.87)	3.13	2.27	0.81 (0.72, 0.91)
PPROM	8.32	11.56	1.02 (0.93, 1.13)	8.32	8.65	0.89 (0.83, 0.95)
Chorioamnionitis	1.68	2.28	1.06 (0.85, 1.34)	1.68	1.80	0.93 (0.80, 1.08)
SGA birth	7.11	7.63	1.02 (0.91, 1.15)	7.11	7.27	1.00 (0.93, 1.07)

Abbreviation: Vac, vaccinated with quadrivalent or trivalent influenza vaccine in successive pregnancies; No vac, not vaccinated with influenza vaccine in successive pregnancies; RR, relative risk; PE/eclampsia, preeclampsia or eclampsia; PTB, preterm birth; PPROM, preterm premature rupture of membranes; SGA, small for gestational age

[a] Analyses were adjusted for gestational age at index date, maternal age, race and ethnicity, maternal education, smoking and alcohol use during pregnancy, pre-pregnancy body mass index, gestational weight gain, timing of prenatal care initiation/number of prenatal care visits, maternal comorbidities (chronic hypertension, diabetes mellitus, renal disease, and autoimmune disease), history of adverse perinatal outcomes in a prior pregnancy, month of conception, year of pregnancy, receipt of influenza vaccine during the interpregnancy period, receipt of other vaccines during pregnancy, and Vaccine Safety Datalink

[b] Estimate was not available due to small sample size and model convergence issue

eFigure 1. Cohort Composition: Vaccine Safety Datalink (VSD), 2004-2018

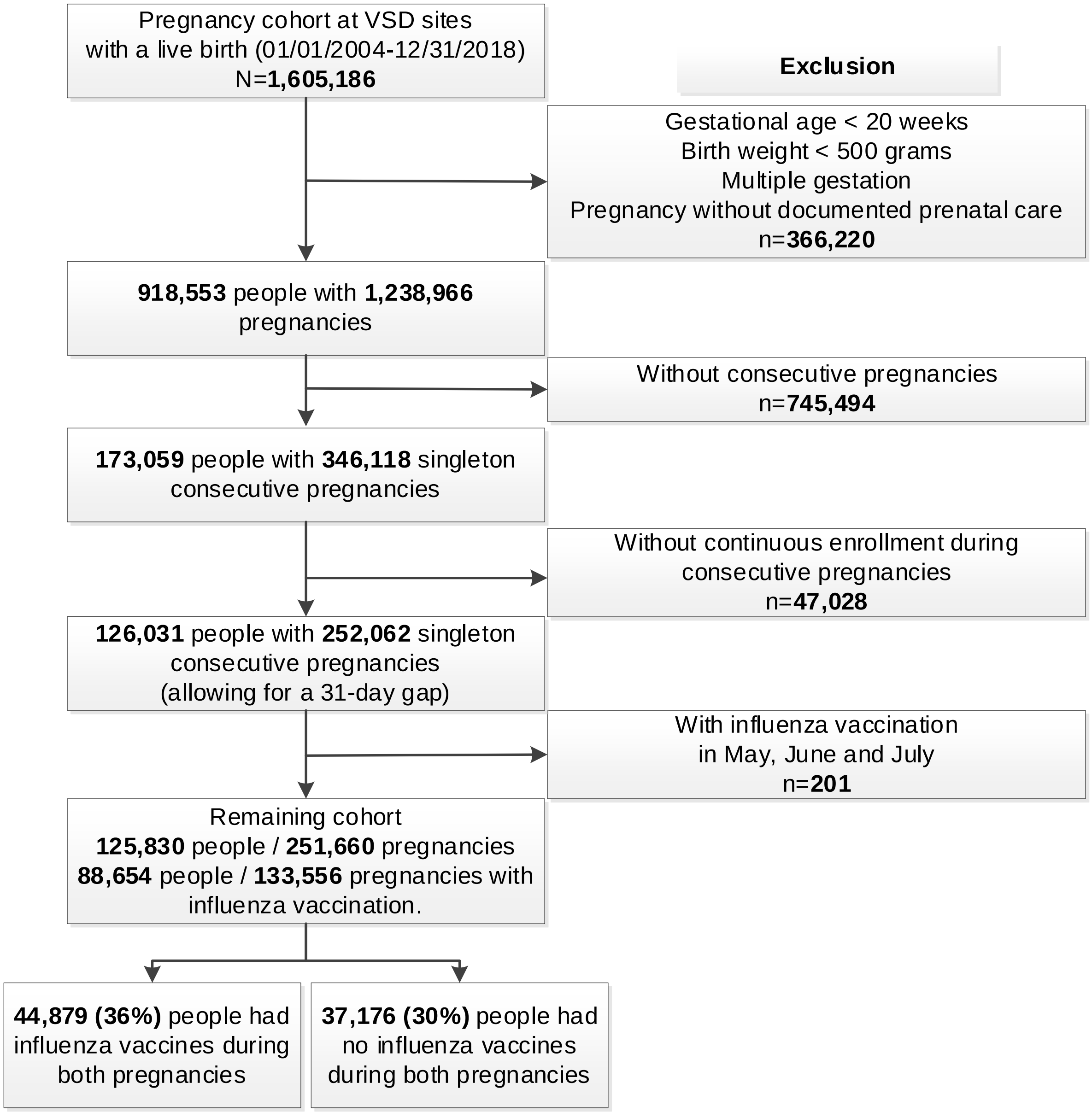

Figure 2. Distribution of gestational week at index date in the influenza vaccinated group and unvaccinated group: Vaccine Safety Datalink, 2004-20

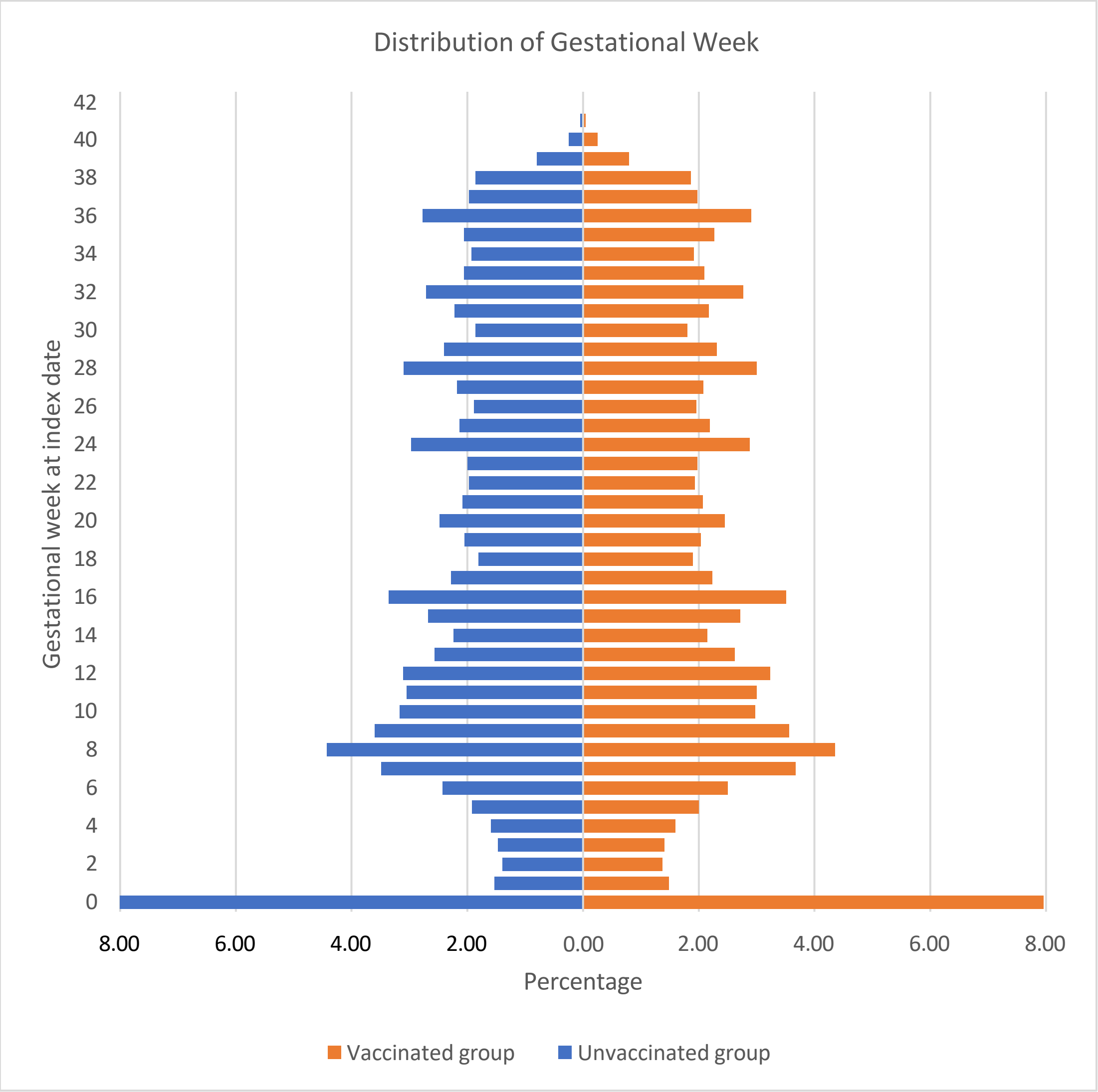

Supplement: Supplement 1. — eTable 1. Total Sample Size Required to Achieve 80% Power for Various Detectable Relative Risks and Outcome Rates, Assuming the Sizes of the Vaccinated and Unvaccinated Groups Are Equal and a Type 1 Error Rate of 0.05. eTable 2. List of Outcomes of Interest and Corresponding ICD-9/ICD-10 Codes eTable 3. Rates and Relative Risks of Adverse Outcomes in Second Pregnancy Based on Maternal Vaccination Status in Successive Pregnancies eTable 4. Test for Interaction Between Vaccination Status and Interpregnancy Interval eTable 5. Incidence and Relative Risk of Adverse Perinatal Outcomes in Second Pregnancy Among People With Influenza Vaccination in Successive Pregnancies Versus People With No Influenza Vaccination in Successive Pregnancies, by Vaccine Type: Vaccine Safety Datalink, 2004-2018 eFigure 1. Cohort Composition: Vaccine Safety Datalink, 2004-2018 eFigure 2. Distribution of Gestational Week at Index Date in the Influenza Vaccinated Group and Unvaccinated Group: Vaccine Safety Datalink, 2004-2018 [file jamanetwopen-e2434857-s001.pdf]
