# Supplementary material for: Safety of the Seasonal Influenza Vaccine in 2 Successive Pregnancies
Source: JAMA Netw Open. 2024 Sep 19;7(9):e2434857. doi: 10.1001/jamanetworkopen.2024.34857 (PMC11413712; doi:10.1001/jamanetworkopen.2024.34857)
Supplement: Supplement 2. — Data Sharing Statement [file jamanetwopen-e2434857-s002.pdf]

Data Sharing Statement

Getahun. Safety of the Seasonal Influenza Vaccine in 2 Successive Pregnancies. *JAMA Netw Open*. Published September 19, 2024. doi:10.1001/jamanetworkopen.2024.34857

Data
Data available: No

Additional Information
Explanation for why data not available: The data that support the study conclusions are unavailable for public access. Guidelines on how to access VSD data through a sharing program administered by the National Center for Health Statistics Research Data Center (NCHSRDC) are provided here: https://www.cdc.gov/vaccinesafety/ensuringsafety/monitoring/vsd/data-sharing-guidelines.html and are subject to change.
